# Metagenomic Reconstruction of Key Anaerobic Digestion Pathways in Municipal Sludge and Industrial Wastewater Biogas-Producing Systems

**DOI:** 10.3389/fmicb.2016.00778

**Published:** 2016-05-24

**Authors:** Mingwei Cai, David Wilkins, Jiapeng Chen, Siu-Kin Ng, Hongyuan Lu, Yangyang Jia, Patrick K. H. Lee

**Affiliations:** School of Energy and Environment, City University of Hong KongHong Kong, China

**Keywords:** biogas, anaerobic digestion, metagenomes, municipal sludge, industrial wastewater

## Abstract

Anaerobic digestion (AD) is a microbial process widely used to treat organic wastes. While the microbes involved in digestion of municipal sludge are increasingly well characterized, the taxonomic and functional compositions of AD digesters treating industrial wastewater have been understudied. This study examined metagenomes from a biogas-producing digester treating municipal sludge in Shek Wu Hui (SWH), Hong Kong and an industrial wastewater digester in Guangzhou (GZ), China, and compared their taxonomic composition and reconstructed biochemical pathways. Genes encoding carbohydrate metabolism and protein metabolism functions were overrepresented in GZ, while genes encoding functions related to fatty acids, lipids and isoprenoids were overrepresented in SWH, reflecting the plants’ feedstocks. Mapping of genera to functions in each community indicated that both digesters had a high level of functional redundancy, and a more even distribution of genera in GZ suggested that it was more functionally stable. While fermentation in both samples was dominated by *Clostridia*, SWH had an overrepresentation of *Proteobacteria*, including syntrophic acetogens, reflecting its more complex substrate. Considering the growing importance of biogas as an alternative fuel source, a detailed mechanistic understanding of AD is important and this report will be a basis for further study of industrial wastewater AD.

## Introduction

Anaerobic digestion (AD) is a biological decomposition process widely used in municipal wastewater treatment plants (WWTPs). Globally, biogas-producing AD processes are gaining attention because they can not only degrade organic waste, which reduces water quality and poses a danger to public health if not properly treated (Sahlstrom, 2003; Bibby and Peccia, 2013), but also provide a renewable source of energy in the form of methane (biogas; Angelidaki and Ellegaard, 2003; Weiland, 2003; Luo et al., 2013). Despite its widespread use worldwide, the biological mechanisms of AD are still poorly understood, mostly due to the complexity of the microbial communities involved (Wagner and Loy, 2002; Nelson et al., 2011). Thus, detailed studies of the composition of AD microbial consortia and their metabolic functions are required. An improved understanding of AD could enhance the efficiency of carbon recovery from waste streams, contributing to the global goal of turning WWTPs into sustainable systems (Jetten et al., 1997).

Identifying the microorganisms in AD systems has traditionally been accomplished by the construction of 16S rRNA gene clone libraries followed by Sanger sequencing (Riviere et al., 2009), which in recent years has been replaced by high-throughput next-generation sequencing of 16S rRNA gene amplicons (Schluter et al., 2008; Werner et al., 2011). However, both these methods focus on taxonomic identification, and the metabolic pathways present can be determined only indirectly. Moreover, these methods can introduce biases as a PCR amplification step is required. Shotgun metagenomic sequencing, which directly sequences the extracted DNA, can provide more detailed information on the identity of the microbes and their metabolisms as well as other biological information including novel genes (Ferrer et al., 2005a,b). In addition to these advantages, metagenomic sequencing is gaining importance in the study of microbial communities because the decreasing cost and increasing sequencing depth have enabled high-resolution analysis of complex environmental samples (Qin et al., 2010; Fierer et al., 2012) such as sea water (Tang et al., 2013), soils (Fierer et al., 2012), human gut (Qin et al., 2010), and freshwater (Breitbart et al., 2009). Metagenomics was first applied to AD in 2008 with the analysis of a German full-scale biogas plant treating farm waste (Schluter et al., 2008). Several further studies have since examined AD metagenomes, with the focus mainly on taxonomy and gene-centric functional analyses (Wirth et al., 2012; Wang et al., 2013; Wong et al., 2013; Yang et al., 2014). Recently, metagenomic analysis has shifted toward reconstructing important metabolic pathways and genomes present in AD systems (Li et al., 2013).

Of the AD metagenomes analyzed to date, samples have been obtained from full-scale biogas plants treating farm waste (Schluter et al., 2008), industrial (Wang et al., 2013) and municipal (Wong et al., 2013; Yang et al., 2014) sludge digesters, and lab-scale reactors (Wirth et al., 2012; Li et al., 2013). However, little analysis has yet been conducted of full-scale AD systems treating high-strength industrial wastewater. While municipal sludge digesters are important, AD systems treating high-strength wastewater should not be neglected because a substantial volume of industrial wastewater is generated every year. In China alone, the discharge of industrial wastewater was 6.9 × 10^10^ metric tons in 2012 and estimated to be 7.8 × 10^10^ metric tons in 2015, accounting for as high as 35% of total national wastewater discharge (Feng et al., 2015). This study aimed to determine whether and how the major AD processes and the taxonomic groups performing them differ in a high-strength industrial wastewater system compared to better-studied municipal sludge systems. We obtained metagenomes from one system of each type and reconstructed and compared their key AD metabolic pathways. The major taxonomic groups performing these processes were determined and compared between the two systems, and pathways with particular functional significance analyzed. The redundancy of organisms in the major pathways of the multi-step AD process was also examined.

## Materials and Methods

### Sample Descriptions

Anaerobic digestion samples were collected from a full-scale industrial digester treating high-strength wastewater located in Guangzhou, China (hereafter abbreviated ‘GZ’), and a municipal digester treating sludge located in Shek Wu Hui, Hong Kong (‘SWH’). Detailed descriptions of the digester systems and sample collection procedures have previously been reported (Wilkins et al., 2015a). Briefly, GZ treats 0.7 million liters per day of high-strength industrial wastewater from beverage manufacturing with a retention time of 0.5 days. SWH treats 0.45 million liters per day of municipal sewerage sludge with a retention time of 23 days. The daily methane production and operating temperature are higher for SWH. Operating conditions and measured parameters are provided in Supplementary Table S1.

### DNA Extraction and Illumina Sequencing

Genomic DNA was extracted using the PowerSoil DNA Isolation Kit (Mo Bio Laboratories, Carlsbad, CA, USA) as described previously (Lu et al., 2013). 6 μg of extracted DNA for each sample was used for library construction using the Illumina paired-end DNA sample preparation kit according to the manufacturer’s instructions. The prepared libraries were sequenced using paired-end 100 bp reads on an Illumina HiSeq 2000 according to manufacturer’s recommendations by BGI-Hong Kong. After removing reads containing ‘N’ and adapters, a total of ∼16 Gb of paired-end reads (90 bp in length) were generated for both samples (∼8 Gb for each sample). Sequencing reads from this study have been deposited in Metagenome Rapid Annotation using Subsystem Technology (MG-RAST^1^) with accession numbers 4560350.3 (GZ) and 4560351.3 (SWH).

### Taxonomic and Functional Annotation of Metagenomes

As recommended by the online metagenome analysis tool MG-RAST version 3.0 (Mason et al., 2014; Wilbanks et al., 2014), the reads were not assembled or filtered before submission for taxonomic and functional analyses. Read paired ends were merged prior to analysis according to the instructions provided by MG-RAST. Artificial replicate sequences and irrelevant sequences (e.g., plant, human, or mouse) were removed automatically by MG-RAST and low-quality sequences were filtered out using default settings. The read sets were normalized by random subsampling to 3.9 Gb, and rarefaction curves constructed to assess whether sequencing effort was sufficient to capture the majority of taxonomic diversity.

Reads were taxonomically annotated by comparison with the M5NR database using sBLAT (Kent, 2002). M5NR is a non-redundant (nr) protein database (Meyer et al., 2008) comprising the NCBI GenBank (Benson et al., 2008), SEED (Overbeek et al., 2005), KEGG (Kanehisa and Goto, 2000), IMG terms (Markowitz et al., 2008), eggNOGs (Jensen et al., 2008), and Uniprot (Magrane and UniProt Consortium, 2011) databases. To identify genes and their functions, the reads were additionally annotated via sBLAT (Kent, 2002) searches against the COG and SEED gene databases, both of which organize genes into nested hierarchies of groups (COG categories and SEED subsystems) with related functional roles. For both the taxonomic and functional annotations, the best hit was accepted as the annotation for that read, and only read alignments ≥ 25 nucleotides with similarity to the reference database ≥ 60% and *E*-value ≤ 1 × 10^-5^ were retained (Tang et al., 2013; Wong et al., 2013; Mason et al., 2014). Taxon, COG category and SEED subsystem counts were normalized by dividing by the total number of hits in each metagenome (Mackelprang et al., 2011; Tang et al., 2013).

### Reconstruction of Metabolic Pathways

Anaerobic digestion of organic compounds has four major steps: hydrolysis, acidogenesis, acetogenesis, and methanogenesis (Wirth et al., 2012). Representative pathways for the acidogenesis, acetogenesis, and methanogenesis steps of AD and for denitrification were constructed according to recently published articles (Francis et al., 2007; Agler et al., 2011) with reference to databases including KEGG, MetaCyc (Caspi et al., 2014), and BRENDA (Scheer et al., 2011). These pathways included the reaction steps producing major intermediate compounds and the enzymes that catalyze these steps. The relative abundances of the top five genera from each metagenome associated with the last step in the formation of major intermediates in acidogenesis, acetogenesis, and methanogenesis, and the top five genera from each metagenome associated with the major enzymes in the methanogenesis and denitrification pathways were mapped to the respective pathways. The Pielou evenness index was calculated for the top genera associated with major intermediates and for all genera in each digester using the R package vegan^2^.

To gain insight into the functions and organisms involved in hydrolysis and to examine the samples’ taxonomic and functional profiles more generally (Smith et al., 2012; Luo et al., 2013; Tang et al., 2013), Statistical Analysis of Metagenomic Profiles (STAMP, version 2.0; Parks and Beiko, 2010) was used to compare the abundances of taxa, COG categories and SEED subsystems between the samples. Detailed information regarding the method can be found in the manual^3^. Significant differences were identified with the two-sided Fisher’s exact test (Fisher, 1958), with 0.95 confidence intervals determined by the Newcombe-Wilson method (Newcombe, 1998). Storey’s False Discovery Rate (FDR; Storey and Tibshirani, 2003) was used to correct for multiple comparisons, and results with a *q*-value (corrected *p*-value) <0.05 retained.

### Assembly and Analyses of Contigs

Although the primary taxonomic and functional annotation was performed with unassembled reads as recommended by MG-RAST, contigs were assembled from each metagenome to identify longer sequences present in both samples and to perform thorough searches for key marker genes. Raw sequence reads were filtered and trimmed using Fastq-Mcf^4^ with default settings. The filtered reads were merged and converted to FASTA format using ‘fqwfa’ (Ruby et al., 2013) followed by *de novo* assembly with IDBA_UD version 1.1.1 using the options ‘–step 10,’ ‘–mink 20,’ ‘–maxk 100,’ and ‘–min_contig 800’ (Peng et al., 2012). Contigs >1200 bp in length were retained.

To identify long sequences shared between the samples, contigs from both samples were compared pairwise with BLASTN. Pairwise hits with an alignment length ≥3,000 bp and sequence identity ≥ 95% were retained and annotated via a BLAST search against the NCBI nr nucleotide database. The assembled contigs were also annotated in MG-RAST by searching against the M5NR, COG, and SEED databases as described above. Custom databases of protein sequences for all enzymes involved in methanogenesis were constructed from the KEGG database and contigs were compared with these databases to detect key methanogenesis genes.

### Comparisons with Other Metagenomes

Metagenomes representing other AD systems (Supplementary Table S2) were submitted to MG-RAST for comparison with this study’s metagenomes. Reads from the other metagenomes that matched reads from this study with an *E*-value ≤ 1 × 10^-3^ were retained (Smith et al., 2012; Tang et al., 2013). To examine changes in the SWH community over time, functional and taxonomic profiles of two metagenomes obtained from SWH as part of a different study (Yang et al., 2014) were obtained. The operating conditions of the digester did not change significantly between the two studies. All taxon and function abundances were proportionally normalized as described above.

## Results

### Overview of the Metagenomes

Following read quality control, a total of 14,949,014 and 20,124,753 reads with average lengths of 152 ± 24 bp and 152 ± 21 bp in GZ and SWH, respectively, were retained (Supplementary Table S3). After annotation, 32.3% of GZ and 48.3% of SWH reads attracted matches to the M5NR database, and 4,280,812 and 5,774,507 reads containing functional genes were identified in GZ and SWH, respectively (Supplementary Table S3). A wide range of sequencing depths, from 600 Mb (Wilbanks et al., 2014) to 3 Gb (Yang et al., 2014), have previously been used in metagenomic analyses. Rarefaction curves were asymptotic for both metagenomes at the 3.9 Gb subsampling depth (Supplementary Figure S1), showing this depth is sufficient to cover the majority of species richness.

### Taxonomic Composition of the Metagenomes

**FIGURE 1:**
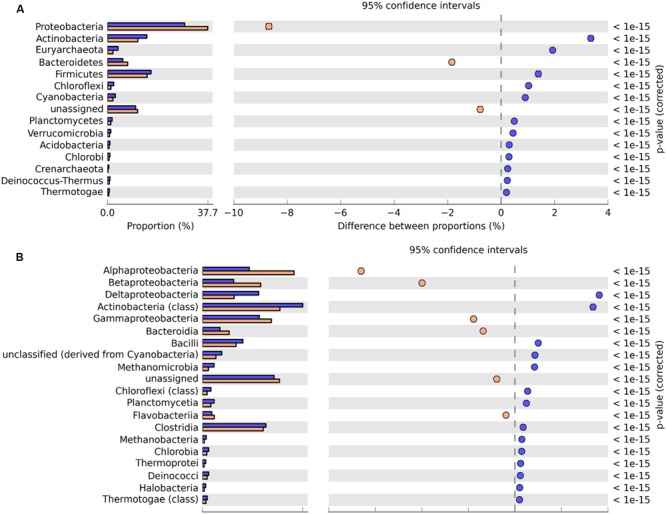
**Significant differences in microbial communities at the **(A)** phylum and **(B)** class level between GZ (blue) and SWH (yellow).** Significance was determined by Fisher’s exact test with Story’s FDR correction for multiple comparisons, with corrected *p* < 0.05 considered significant. Only taxa with difference between proportions >0.2 (i.e., considered large effect) are shown.

The taxonomic composition of the metagenomes was determined from the annotation of reads against the M5NR database in MG-RAST (Meyer et al., 2008). At the domain level, the majority of reads were assigned to *Bacteria* (79.5% for GZ; 83.0% SWH), followed by *Archaea* (7.4% GZ; 4.0% SWH), and *Eukaryota* (1.0% GZ; 0.7% SWH). Within the *Bacteria*, the most abundant phyla were *Proteobacteria, Firmicutes, Actinobacteria*, and *Bacteroidetes* in both samples. STAMP analysis identified a significant overrepresentation of *Firmicutes, Actinobacteria*, and *Euryarchaeota* in GZ while *Proteobacteria* and *Bacteroidetes* were overrepresented in SWH (**Figure 1A**).

Class abundances were highly correlated between the two samples (*R*^2^ = 0.88). In both samples, *Actinobacteria* (class) were highly abundant, followed by *Clostridia, Bacilli*, and classes of the *Proteobacteria*. The *alpha*-, *beta*-, and *gamma*-*Proteobacteria* were significantly overrepresented in SWH, while *delta-Proteobacteria*, which contains acetogenic bacteria that interact syntrophically with methanogens (Lopez-Garcia and Moreira, 2006; Sousa et al., 2009), were overrepresented in GZ (**Figure 1B**). These differences are likely due to differences in the feedstocks and/or operating conditions (e.g., retention time and temperature, Supplementary Table S1).

One thousand nine hundred thirty-nine genera were identified in GZ and 2,554 genera in SWH, of which 1,612 were shared between the metagenomes. Apart from high representations of *Streptomyces* in GZ and *Mycobacterium* in SWH (Supplementary Figure S2), the major genus in both metagenomes was *Clostridium* which formed a similar proportion of both samples (GZ 2.7%; SWH 2.6%). The abundance of minor genera varied greatly between the two metagenomes. Pathogens including *Mycobacterium* and *Burkholderia* were significantly more abundant in SWH, whereas bacterial genera associated with acetogenesis (e.g., *Syntrophobacter*) were overrepresented in GZ (Supplementary Figure S2).

*Euryarchaeota*, which includes all known methanogens (Li et al., 2013), was the most abundant archaeal phylum in both samples, followed by *Crenarchaeota, Thaumarchaeota, Korarchaeota*, and *Nanoarchaeota* (Supplementary Table S4). Within the *Euryarchaeota*, the methanogenic classes *Methanomicrobia* and *Methanobacteria* dominated both metagenomes, while at the order level *Methanomicrobiales* was most abundant followed by *Methanosarcinales* and *Methanobacteriales* (Supplementary Table S4). At the genus level, *Methanosarcina* was prevalent in both samples (GZ 9.3% of all *Archaea*; SWH 8.4%) while *Methanothermobacter* were abundant in GZ (GZ 4.9%; SWH 3.6%) and *Methanospirillum* (GZ 3.7%; SWH 6.8%), *Methanobrevibacter* (GZ 4.2%; SWH 5.4%), and *Thermococcus* (GZ 4.9%; SWH 5.4%) in SWH. *Methanosaeta* (GZ 2.7%; SWH 2.6%) were present in low abundance in both samples.

### Functional Composition of the Metagenomes

To obtain metabolic function profiles for the two metagenomes, all reads were annotated via sBLAT search against the COG and SEED databases. In both samples, genes encoding functions from metabolism-related COG categories were dominant, representing more than 45% of the total hits within each sample. Within the metabolism COG category, more than 65% of reads in both samples were annotated with sequences in the categories carbohydrate transport and metabolism, lipid transport and metabolism, energy production and conversion, and amino acid transport and metabolism. A high proportion of genes related to these functions has been previously reported in metagenomes (Li et al., 2013) and metaproteomes (Wilmes et al., 2008) from AD systems and may indicate a metabolism suitable for anaerobic microbial reactions. STAMP analysis indicated that genes related to carbohydrate transport and metabolism were overrepresented in GZ, while lipid transport and metabolism genes were overrepresented in SWH (Supplementary Figure S3).

**FIGURE 2:**
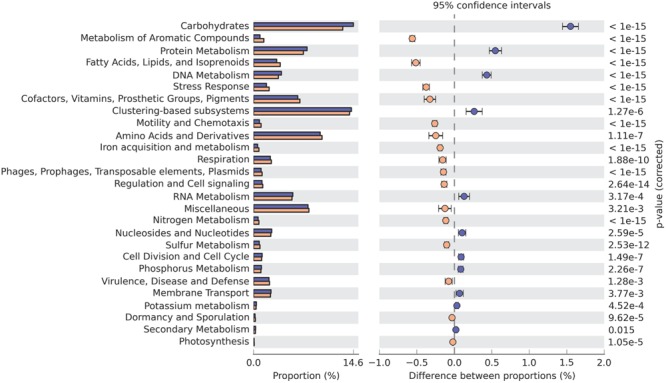
**SEED subsystems (level 1) with significantly different abundances in the GZ (blue) and SWH (yellow) metagenomes.** Significance was determined by Fisher’s exact test with Storey’s FDR correction for multiple comparisons, with corrected *p* < 0.05 considered significant.

Level 1 (broadest level) SEED subsystems such as clustering-based systems and carbohydrates metabolism were relatively abundant in both samples, accounting for almost 15% of each. The amino acids and derivatives metabolism and protein metabolism subsystems were also prevalent. As with the COG results, most of these abundant subsystems were related to the degradation of organic matter. Genes from carbohydrates metabolism and protein metabolism subsystems were overrepresented in GZ, whereas genes from fatty acids, lipids and isoprenoids subsystems were more abundant in SWH (**Figure 2**). In both anaerobic wastewater and sludge metagenomes, a number of genes related to nitrogen metabolism and to respiration were also detected, corroborating a previous finding (Ye et al., 2012).

Among level 4 (gene product) SEED subsystems, the most prevalent genes in both samples were those encoding glycosyl transferase, followed by decarboxylase. Glycosyltransferase had the largest difference between proportions in the two samples of all level 4 subsystems, being highly and significantly overrepresented in GZ (Supplementary Figure S4).

### Reconstruction of Functional Pathways

**FIGURE 3:**
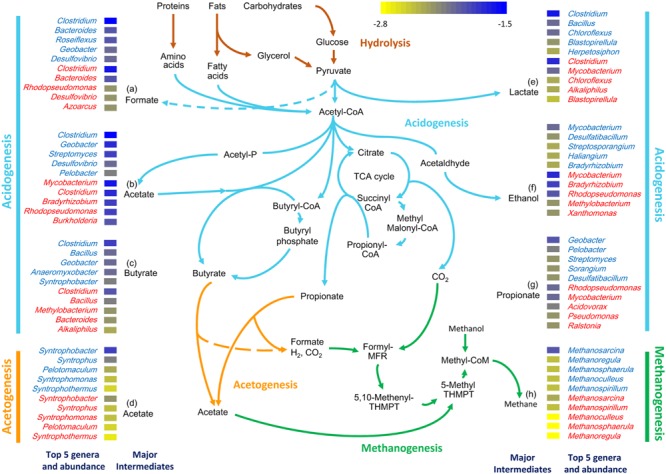
**Genera in the GZ (genus name in blue) and SWH (genus name in red) metagenomes mapped to each major AD step (acidogenesis, acetogenesis, and methanogenesis).** The five most abundant genera mapped to the last step in the formation of the major intermediates are shown (labeled with a letter). The log_10_-normalized relative abundance of each genus is indicated by the colored tile beside its name.

To determine the major genera involved in AD in each sample, the abundances of genera associated with the last step in the formation of major intermediate products were examined. Genera contributing to the formation of acetate were dominant in both samples at the acidogenesis stage (**Figure 3**). A high abundance of genera capable of ethanol fermentation (e.g., *Bradyrhizobium*) was found in the sludge metagenome (SWH), while a high proportion of genes were assigned to the genera responsible for formate formation (e.g., *Clostridium* and *Bacteroides*) in the wastewater metagenome (GZ). In addition, genes from *Clostridium* were most frequently detected in the acidogenesis process in both samples, consistent with the high abundance of *Clostridium* in GZ and SWH (**Figure 3**).

Unlike acidogenesis, acetogenesis is a rate-limiting step as the conversion of long-chain volatile fatty acids (VFAs) to acetate, formate, CO_2_, and H_2_ is endothermic under standard conditions and limited by factors including H_2_ partial pressure and substrate concentration (Müller et al., 2010). Changes in these metabolite concentrations therefore have a large effect on the overall AD rate (McInerney et al., 2009). Acetogenesis was dominated in both metagenomes by the *delta*-proteobacterial genus *Syntrophobacter*, members of which can convert butyrate/propionate to acetate (Müller et al., 2010). Methanogenesis in both samples was likely contributed by the dominant genus *Methanosarcina*.

Overall, acidogenesis genera were less abundant than acetogenesis and methanogenesis genera in both digesters (Supplementary Table S5). However, for the formation of acetate as an intermediate product of acidogenesis, the relative abundances of the top genera were similar in both samples (**Figure 3**). Furthermore, the relative abundances of the single major acetogenic [e.g., *Syntrophobacter* (1.4%)] and methanogenic [e.g., *Methanosarcina* (1.1%)] genera were similar to those of the less evenly distributed major acidogenic genera [e.g., *Clostridium* (1.3–2.7%) and *Geobacter* (0.9–2.0%)] in GZ. Meanwhile, genera were overall more evenly distributed in GZ (Supplementary Table S5).

The methanogenesis and denitrification pathways were selected for more detailed pathway reconstructions in which all major enzymes were considered. Compounds including H_2_/CO_2_, acetate, and some C1 compounds (e.g., formate, methanol, dimethylamine, and methanethiol) can serve as substrates for methanogenesis (Francis et al., 2007; Li et al., 2013). Both the hydrogenotrophic (H_2_/CO_2_ to methane) and acetoclastic (acetate to methane) pathways were detected in both metagenomes, though genes encoding enzymes in the acetoclastic pathway (EC 2.7.2.1, EC 2.3.1.8, EC 6.2.1.1, and EC 2.3.1.-) were more abundant than those in the hydrogenotrophic pathway (EC 1.2.99.5, EC 2.3.1.101, EC 3.5.4.27, EC 1.5.98.1, and EC 1.5.98.2) in both samples (Supplementary Figure S5). Within the acetoclastic pathway, genes encoding enzyme EC 6.2.1.1 (responsible for the formation of acetyl-CoA from acetate) were more abundant than EC 2.7.2.1 (acetate kinase) and EC 2.7.1.8 (phosphate acetyltransferase) in both metagenomes (Supplementary Figure S5). Although methanol-utilizing methanogens (e.g., *Methanosphaera stadtmanae* and *Methanosarcina barkeri*) were found in both metagenomes, no sequence encoding enzymes *mtaA* (coenzyme M-binding methyltransferase) or *mtaB* (methanol-binding methyltransferase) was detected in reads or contigs from either metagenome, possibly due to incomplete sequencing.

As expected under anaerobic conditions, genes encoding enzymes involved in nitrification (e.g., ammonia monooxy genase, EC 1.14.99.39 and EC 1.7.2.6) were either undetected or at low abundance in both GZ and SWH (Supplementary Figure S6). In contrast, a high abundance of enzymes related to denitrification (nitrate reductases, EC 1.7.99.4, EC 1.7.2.4, and EC 1.7.2.1; nitrous oxide reductase, EC 1.7.2.5) was detected (Supplementary Figure S6). In addition, a high proportion of reads in both samples were assigned to nitrite reductase (EC 1.7.1.4) and nitrogenase (EC 1.18.6.1) genes (Supplementary Figure S6). Genes for hydrazine oxidoreductase (EC 1.7.99.8), an enzyme involved in anaerobic ammonia oxidation (anammox), were searched for by BLASTP in contigs from both samples but not found. As previously reported (Francis et al., 2007), both bacteria and archaea contributed enzymes to the denitrification pathway.

### Comparison with Other Metagenomes

**FIGURE 4:**
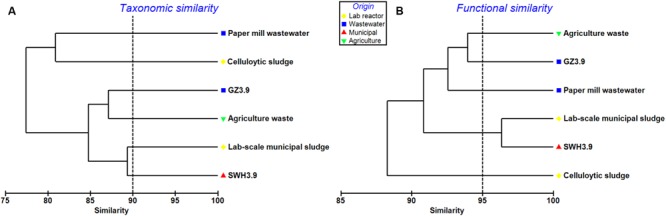
**Clustering of the GZ and SWH metagenomes with other AD metagenomes at **(A)** the genus level, representing taxonomic similarity and **(B)** level 3 SEED subsystems, representing functional similarity.** Metagenomes were clustered based on the Bray–Curtis similarity with square-root transformed abundances using PRIMER software. Dashed lines are inserted for visualizing the similarity comparisons.

Four additional AD metagenomes were selected for comparison with GZ and SWH (Supplementary Table S2). The GZ metagenome was taxonomically and functionally most similar to that of a German production-scale biogas plant fed with a mixture of crops (∼98%) and chicken manure (∼2%), but was distinct from those treating cellulose sludge and lab-scale municipal sludge (**Figure 4**). SWH shared a high degree of similarity, especially among functional genes (similarity >95%), with a metagenome from a lab-scale anaerobic sludge reactor (**Figure 4**; Lv et al., 2014). Two metagenomes from AD systems treating paper mill wastewater and cellulolytic sludge were very dissimilar from the metagenomes in this study.

To investigate temporal variability in taxa and functions, we examined two additional metagenomes also from SWH but collected at different times for a different study (Yang et al., 2014). The two samples used for comparison were collected in September 2011 and March 2012, while the sample in this study was collected in November 2011. A comparison of the taxonomic and functional profiles indicated shifts in the dominant microbial community members present (Supplementary Figure S7), even between the two samples from the previous study that were sequenced using the same method. Smaller changes were observed in the functional gene profiles, especially for essential functions (e.g., transcription and DNA replication).

## Discussion

Anaerobic digestion has been widely applied in the treatment of municipal sludge (Riviere et al., 2009; Yang et al., 2014) and industrial wastewater (Rajeshwari et al., 2000) as this technology decomposes organic waste while simultaneously producing biogas (Angelidaki and Ellegaard, 2003; Weiland, 2003; Luo et al., 2013). However, previous AD studies have mainly focused on the phylogenetic diversity of municipal digesters treating waste sludge from secondary treatment (Yang et al., 2014) or farm waste (Schluter et al., 2008). The taxonomic and functional composition of AD digesters treating high-strength industrial wastewater have not been extensively studied, especially those taxa and functions involved with the major AD steps (Wagner and Loy, 2002; Nelson et al., 2011). Given the vast and increasing volume of high-strength industrial wastewater produced worldwide, the application of AD to wastewater is likely to grow. Therefore, it is important to thoroughly understand the microbiology involved in the anaerobic treatment of wastewater and how it differs from the more common sludge digesters. We analyzed metagenomes (i.e., covering both taxonomy and metabolic functions) from an industrial wastewater AD system and a municipal system treating sludge to reconstruct the major AD biochemical processes and examine how they differ between these two digester types.

In this study, we chose not to assemble the reads before annotation, as low-coverage sequences might be excluded by an assembler (Howe et al., 2014) and bias the taxonomic profile against rare taxa. Unlike some studies using unassembled reads (Fierer et al., 2012), here we used merged paired end reads for more reliable annotation results. However, the protein annotation rates (53.4% for GZ and 48.3% for SWH) were similar to reported rates for unmerged reads (42.7–56.1%; Fierer et al., 2012; Wirth et al., 2012). We also assembled contigs to provide a basis for phylogenetic comparison between the metagenomes, although these were not used to generate taxonomic and functional abundances. The annotation rates in these contigs were < 68%, suggesting incomplete sequencing of genes played some role in the annotation rate but did not account for all unannotated reads. Unannotated reads could also be attributed to the high diversity of the samples (Wilkins et al., 2015a) and/or incomplete databases (Ye et al., 2012).

The presence of functional genes in an AD system should generally be correlated to the substrate it is treating. For example, a metagenome from anaerobic digestion of tannery wastewater found genes assigned to protein metabolism were the most abundant, making up about 15% of the metagenome (Wang et al., 2013). In the metagenome from the GZ digester, which treats carbohydrate-rich beverage wastewater, genes related to carbohydrate transport and metabolism were the most abundant and strongly overrepresented relative to SWH (**Figure 2**; Supplementary Figure S3; Hu et al., 2010). In contrast, functions related to metabolism of lipids and fatty acids were significantly overrepresented in SWH, including the SEED subsystem for fatty acids, lipids, and isoprenoids (**Figure 2**), COG category for lipid transport and metabolism (Supplementary Figure S3B) and genes encoding enzymes from the cytochrome P450 family (Supplementary Figure S4). Cytochrome P450 enzymes catalyze reactions on a broad range of substrates, particularly lipid metabolites such as steroids, eicosanoids, fatty acids, and lipids (Coon et al., 1992). This functional profile of SWH suggests there was a higher lipid content in the sludge being treated there, as expected for a municipal sewerage waste stream. Similarly, the overrepresentation of SEED subsystems related to the metabolism of aromatic compounds (**Figure 2**) reflects the probable presence of polycyclic aromatic hydrocarbons (PAHs) in sewerage waste streams, particularly concentrated in digester sludge (Blanchard et al., 2004).

Genes encoding the cobalt-zinc-cadmium resistance protein CzcA were abundant in both metagenomes. Cobalt-zinc-cadmium resistance protein CzcA is essential for resistance against certain heavy metals (Diels et al., 1995). Its presence suggests heavy metals such as cobalt, zinc and/or cadmium, could be present in both samples. A previous study has shown that heavy metals are released with the decomposition of organic matter (Dong et al., 2013).

The taxonomic composition of the two digesters also reflected the complexity of hydrolytic functions required to process their feedstocks. The phylum *Proteobacteria* was strongly overrepresented in SWH, with the exception of the class *delta-Proteobacteria* (**Figure 1B**). This overrepresentation was quite evenly spread over a large number of *Proteobacteria* genera (data not shown), indicating it was not due to a few exceptional species but rather a systematic difference. Members of the *Proteobacteria* have a broad range of roles in all AD steps except for methanogenesis. In the pathway reconstruction, *Proteobacteria* genera were among the top contributors to the production of all major fermentation products except lactate and to acetogenesis in both digesters (**Figure 3**). Given this broad range of roles in both digesters, the overabundance of *Proteobacteria* in SWH reflects the more complex range of substrates treated by that system, requiring a more diverse repertoire of functions.

Bacteria in both metagenomes were numerically dominated by reads assigned to the genus *Clostridium*, which can ferment a wide variety of carbon sources and produce VFAs and alcohols that serve as substrates for methanogenesis. A high *Clostridium* abundance has been reported in a range of anaerobic reactors (Nelson et al., 2011) including those fed with crops and a mixture of animal manure (Rademacher et al., 2012) or excess sludge (Li et al., 2013), and in our previous amplicon sequencing-based studies of the GZ and SWH digesters (Wilkins et al., 2015b; Jia et al., 2016). In this study, the class *Clostridia* was slightly overrepresented in the GZ metagenome (**Figure 1**). Mapping of genera to the formation of AD intermediates found that *Clostridium* spp. were the main fermenters forming four of the six major acidogenesis products (formate, acetate, butyrate, and lactate; **Figure 3**), consistent with their reported versatility in AD fermentation (Li et al., 2011). Members of the *Clostridiales* also have roles in initial hydrolysis (Moon et al., 2011; Dassa et al., 2014) and syntrophic acetate oxidation (SAO; Müller et al., 2013). However, it is notable that in our previous study of enrichment cultures inoculated from both digesters *Clostridium* abundance increased in tandem with the methanogen genus *Methanobacterium* (Jia et al., 2016); in this study, the class *Methanobacteriales* was likewise slightly overrepresented in GZ (Supplementary Table S4). SAO bacteria, including *Clostridium ultunense* (<0.1% in both samples; Müller et al., 2013), oxidize acetate to provide H_2_ and CO_2_ to syntrophic partner methanogens such as *Methanobacterium* (Zinder and Koch, 1984), and it is possible that at least some methane production from acetate in the digesters proceeds via this route. As SAO bacteria likely oxidize acetate via the reversible Wood–Ljungdahl pathway (reductive acetyl-CoA pathway; Lee and Zinder, 1988) also used in other AD processes, e.g., methanogenesis, the presence or absence of this route cannot be determined by the presence or absence of marker genes.

As expected for a sewage waste stream, SWH contained a high abundance of human-associated pathogen genera such as *Mycobacterium* (*Mycobacterium tuberculosis, M. bovis* and *M. avium* accounted for ∼72% of *Mycobacterium* in both samples) and *Burkholderia*, although *Mycobacterium* was also unexpectedly abundant in GZ. This high abundance of *Mycobacterium* in both digesters is especially noteworthy as they are unlikely to be important in the degradation of organic compounds under anaerobic conditions (de Oliveira et al., 2010). As removal of *Mycobacterium* at mesophilic temperatures typically requires weeks to months (Sahlstrom, 2003; El-Mashad et al., 2004), the high abundance detected in both systems suggests neither is successful in removing these potential pathogens with the retention time of the systems.

*Methanomicrobiales* was the most abundant archaeal order in both metagenomes (Supplementary Table S4), and contributed most of the abundant genera mapped to the methanogenesis pathway from both digesters (**Figure 3**; Supplementary Figure S5). The orders *Methanosarcinales* and *Methanobacteriales* were also abundant, with both overrepresented in GZ (Supplementary Table S4). Our previous study using amplicon sequencing of the archaeal rRNA gene (Wilkins et al., 2015a) found that *Methanomicrobiales* was dominant in GZ but order *Methanosarcinales* in SWH, while a previous metagenomic study of SWH sludge found an overwhelming (>70%) dominance of *Methanomicrobiales*. These different results may reflect differences in methods, for example the short read length of shotgun metagenomic sequencing leading to conflation of protein sequences from the closely related orders. They could also reflect functional redundancy in the methanogenesis step of AD, i.e., maintenance of the biochemical functions over time independent of variance in taxonomic composition. Such redundancy has been proposed as a feature of higher AD steps, particularly fermentation (Werner et al., 2011), and our previous study of AD enrichment cultures has shown that similar methane yields can be obtained from systems fed with different substrates and containing diverse methanogen communities (Wilkins et al., 2015b). Detailed reconstruction of the methanogenesis pathway (Supplementary Figure S5) found that the functional potential for complete acetoclastic and hydrogenotrophic pathways were present in both metagenomes, with genes encoding enzymes involved in the acetoclastic route dominant in both samples. This agrees with our previous amplicon sequencing study (Wilkins et al., 2015a) and other previous studies that found the acetoclastic pathway tends to be dominant in methanogenic systems (Li et al., 2013; Yang et al., 2014). The time series of SWH metagenomes also suggested greater taxonomic than functional variability, with e.g., the class *Methanomicrobia* increasing 1.4-fold in relative abundance between September 2011 and March 2012 while functional abundances remained relatively constant (Supplementary Figure S7). As different microbes share similar functions (Hashsham et al., 2000), it is quite likely that the presence of functional genes, rather than particular microbial taxa, determines the functional stability of the AD digesters. The presence of the same core functions in each digester contributed by different consortia was also illustrated by the reconstruction of the denitrification pathway, with almost non-overlapping sets of genera responsible for the same biochemical steps in each digester (Supplementary Figure S6).

The abundance of the genus *Methanosaeta* was unusually low in both digesters compared to other reported anaerobic environments, where it is often the most abundant methanogen genus (Ariesyady et al., 2007; Nelson et al., 2011). Our previous amplicon-sequencing based study found the *Methanosaetaceae* to outnumber *Methanosarcinaceae* in both GZ and SWH (Wilkins et al., 2015a). Variation in the relative proportions of *Methanosaeta* and *Methanosarcina* in anaerobic digesters has been linked to operating parameters such as the frequency of substrate feeding (Conklin et al., 2006) and retention time (Ma et al., 2013), as well as biochemical factors including H_2_ partial pressure and the presence of heavy metals (Yilmaz et al., 2014). Pairwise comparison of the contigs assembled from GZ and SWH found that the most similar sequences were mainly methanogens (based on NCBI nr annotation), particularly the strain *Methanosaeta concilii* GP6 (similarity >99%), indicating the two systems shared overall similar methanogen compositions.

In addition to analyzing the distribution of phylogenetic and functional genes as in previous studies (Schluter et al., 2008; Wirth et al., 2012; Wang et al., 2013) or describing the dominant pathways (Li et al., 2013), functional AD pathways were reconstructed and the relative abundances of genera from both metagenomes that may be performing these functions mapped on to these pathways. Genera associated with acidogenesis were less evenly distributed (Supplementary Table S5) than those associated with other steps. This was unexpected, as acidogenesis populations are more reliant on functional redundancy (Werner et al., 2011), while syntrophic bacteria, which are much less abundant than other functional bacteria in AD process, are sensitive to environmental change (McInerney et al., 2009; Werner et al., 2011). The more even distribution of genera in GZ (Supplementary Table S5) indicates that the community should be more functionally stable, as the presence of more parallel pathways provides resilience against fluctuations in substrate loading (Hashsham et al., 2000). This has been experimentally verified in both lab-scale (Hashsham et al., 2000) and full-scale (Werner et al., 2011) reactors, and it has been further shown that evenness rather than richness is the key factor in preserving the functional stability of an ecosystem (Wittebolle et al., 2009). In studies of anaerobic reactors with differing evenness, Werner et al. (2011) found that communities with greater evenness had higher methanogenic activity, suggesting that GZ may have higher methanogenic potential than SWH. Manipulating community evenness (e.g., by transient disturbances; Cabrol et al., 2016) may be a practical strategy for optimizing biogas production.

This study compared municipal wastewater and sludge metagenomes. Despite having different taxonomic profiles, GZ and SWH shared mostly similar potential microbial functions, and of the major functional differences most could be related directly to the digester feedstocks. Mapping of taxa to the major metabolic pathways in AD allowed the major functional taxa in each digester to be determined, and the more even distribution of genera performing major AD functions in GZ suggested a stronger adaptive capability than in SWH (functional stability). We found the metagenome of GZ was similar to that of a production-scale biogas plant both at the phylogenetic and functional level, confirming the biogas-producing potential of industrial wastewater AD. While this study of a single high-strength industrial wastewater AD system may not be globally representative, it strongly suggests that there are major functional differences compared to the better-studied municipal sludge systems that can be directly linked to feedstock, and provides a basis for further investigation of industrial wastewater AD. Future studies should also further explore the AD microbial community with metatranscriptomic and metaproteomic analyses to better understand the metabolic functions.

## Author Contributions

MC analyzed data and wrote the manuscript. DW, JC, S-KN, HL, and YJ analyzed data. PL designed the experiment and wrote the manuscript.

## Conflict of Interest Statement

The authors declare that the research was conducted in the absence of any commercial or financial relationships that could be construed as a potential conflict of interest.

## Footnotes

1 http://metagenomics.anl.gov

2 http://CRAN.R-project.org/package=vegan

3 ttp://kiwi.cs.dal.ca/Software/STAMP

4 http://code.google.com/p/ea-utils/wiki/FastqMcf
