# Supplementary material for: Metagenomic Reconstruction of Key Anaerobic Digestion Pathways in Municipal Sludge and Industrial Wastewater Biogas-Producing Systems
Source: Front Microbiol. 2016 May 24;7:778. doi: 10.3389/fmicb.2016.00778 (PMC4879347; doi:10.3389/fmicb.2016.00778)
Supplement: Supplementary file 1 [file Data_Sheet_1.DOCX]

**Supplementary Material**

**TITLE:** Metagenomic reconstruction of key anaerobic digestion pathways in municipal sludge and industrial wastewater biogas-producing systems

**RUNNING TITLE:** Metagenomes of biogas-producing systems

**AUTHORS:** Mingwei Cai, David Wilkins, Jiapeng Chen, Siu-Kin Ng, Hongyuan Lu, Yangyang Jia, and Patrick K. H. Lee*

**AFFILIATION:** School of Energy and Environment, City University of Hong Kong, Hong Kong, China

***CORRESPONDENT:** B5423-AC1, School of Energy and Environment, City University of Hong Kong, Tat Chee Avenue, Kowloon, Hong Kong; E-mail: [patrick.kh.lee@cityu.edu.hk](mailto:patrick.kh.lee@cityu.edu.hk); Tel: (852) 3442-4625; Fax: (852) 3442-0688

Table S1. Operating conditions and performance of the digesters analyzed in this study^†^

| **Parameter**^a^ | **GZ** | **SWH** |
| --- | --- | --- |
| Daily methane production (m^3^) | 211 | 2,476 |
| Daily vol processed (m^3^) | 700 | 453 |
| Operating temp (°C) | 27-28 | 34-37 |
| pH^b^ | 7.2 ± 0.2 | 7.2 ± 0.1 |
| Retention time (days) | 0.5 | 23 |
| Removal of total solids (%) |  | 47 |
| Removal of volatile solids (%) |  | 11 |
| Removal of chemical oxygen demand (%) | 87.6 |  |

^†^Data are adopted from [Wilkins et al., 2015](#_ENREF_3)

^a^ The digesters were treating feedstocks that originated from a freshwater source. Daily methane production measurements were obtained at ambient temperature and pressure.

^b^ Ranges are given for 1 month of data.

Table S2. Anaerobic digestion metagenomes against which metagenomes from this study were compared.

| **MG-RAST ID** | **Name** | **Description** | **Platform** | **Reference** |
| --- | --- | --- | --- | --- |
| 4524971.3 | Lab-scale municipal sludge | Lab-scale sequencing batch reactor (SBR) fed with municipal sludge and synthetic wastewater/municipal wastewater (10 : 1, v:v) | Illumina | ([Lv et al., 2014](#_ENREF_1)) |
| 4547780.3 | Paper mill wastewater | Anaerobic paper mill wastewater | 454 | Unpublished |
| 4567853.3 | Cellulolytic sludge | Anaerobic thermophilic cellulolytic sludge (lab reactor) | Illumina | ([Xia et al., 2013](#_ENREF_4)) |
| 4567852.3 | Agriculture waste | Biogas fermenter (fed with maize silage, green rye and manure) | 454 | ([Schluter et al., 2008](#_ENREF_2)) |

Table S3. Properties of the two metagenomes.

| **Sample** | **Total sequence after merging**  **(GB)** | **Reads that passed quality control** | **Annotated proteins (%)** |  | **Average length (bp)** |  | **Identified protein features** | **Identified species** |
| --- | --- | --- | --- | --- | --- | --- | --- | --- |
| GZ | 2.3 | 14,022,142 | 53.4 |  | 152 ± 24 |  | 4,280,812 | 7,209 |
| SWH | 3.1 | 20,124,753 | 48.3 |  | 152 ± 21 |  | 5,774,507 | 10,541 |

Table S4. Distribution of *Archaea* in the GZ and SWH metagenomes at the phylum, class and order levels. Relative abundances are expressed as a proportion of the domain *Archaea*.

| **Phylum** | **Class** | **Order** | **GZ** (%) | **SWH** (%) |
| --- | --- | --- | --- | --- |
| *Euryarchaeota* | *Methanomicrobia* | *Methanomicrobiales* | 17.3 | 19.3 |
|  |  | *Methanosarcinales* | 17.3 | 15.5 |
|  |  | *Methanocellales* | 2.0 | 1.6 |
|  | *Methanobacteria* | *Methanobacteriales* | 12.4 | 11.7 |
|  | *Halobacteria* | *Halobacteriales* | 9.9 | 10.8 |
|  | *Methanococci* | *Methanococcales* | 8.6 | 8.7 |
|  | *Thermococci* | *Thermococcales* | 7.8 | 8.6 |
|  | unclassified | unclassified | 3.8 | 3.6 |
|  | *Archaeoglobi* | *Archaeoglobales* | 3.4 | 3.4 |
|  | *Thermoplasmata* | *Thermoplasmatales* | 1.8 | 2.1 |
|  | *Methanopyri* | *Methanopyrales* | 0.7 | 0.6 |
| *Crenarchaeota* | *Thermoprotei* | *Desulfurococcales* | 3.9 | 3.6 |
|  |  | *Thermoproteales* | 3.6 | 3.5 |
|  |  | *Sulfolobales* | 2.5 | 2.6 |
|  |  | *Acidilobales* | 0.3 | 0.3 |
|  | unclassified | unclassified | 0.1 | 0.1 |
| *Thaumarchaeota* | unclassified | *Nitrosopumilales* | 0.5 | 0.4 |
|  |  | *Cenarchaeales* | 0.4 | 0.3 |
|  |  | unclassified | 0.3 | 0.4 |
|  |  | *Nitrososphaerales* | 0.0 | 0.0 |
| *Korarchaeota* | unclassified | unclassified | 0.6 | 0.6 |
| *Nanoarchaeota* | unclassified | unclassified | 0.1 | 0.1 |
| *unclassified* | unclassified | unclassified | 2.8 | 2.5 |

Table S5. Pielou evenness index for the top genera in each step of anaerobic digestion (those genera listed in Figure 3) and for all genera in each sample.

| **Genera** | **Evenness (GZ)** |  | **Evenness (SWH)** |  |
| --- | --- | --- | --- | --- |
| Acidogenesis | 0.89 |  | 0.89 |  |
| Acetogenesis | 0.90 |  | 0.97 |  |
| Methanogenesis | 0.92 |  | 0.93 |  |
| All genera | 0.75 |  | 0.72 |  |


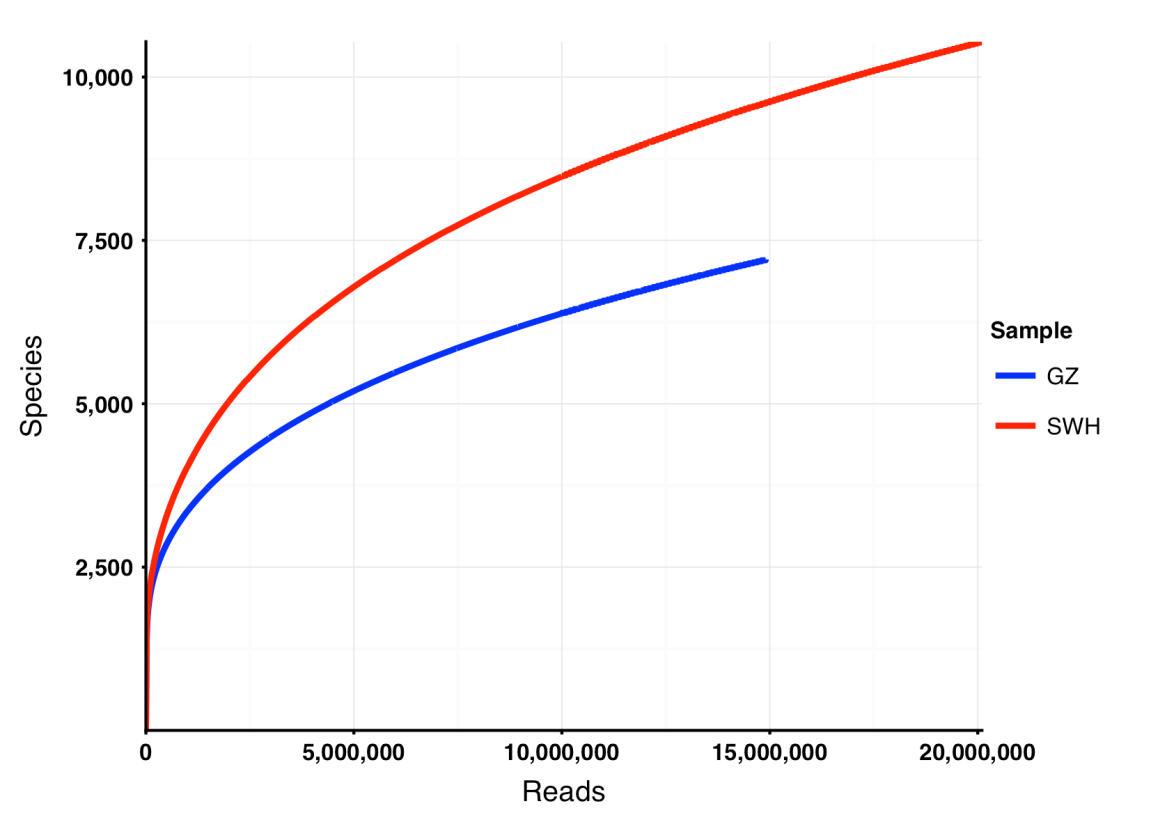


Figure S1. Rarefaction curves for the GZ and SWH metagenomes. Species were annotated against the M5NR database within MG-RAST.


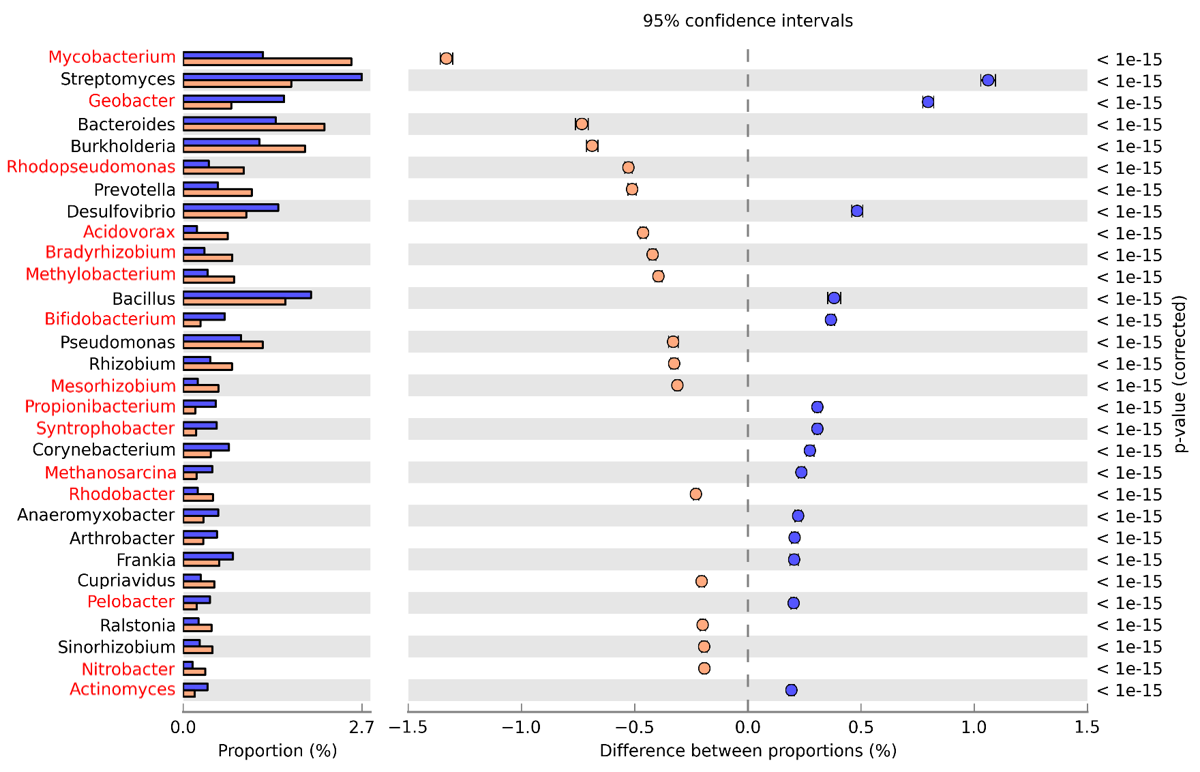


Figure S2. Thirty most abundant genera in GZ (blue) and SWH (yellow). Significance was determined by Fisher’s exact test with Storey’s FDR correction for multiple comparisons, with corrected p < 0.05 considered significant. Genera with a difference between proportions > 0.2 and ratio of proportions > 2 (i.e. considered large effect) are labeled in red.**
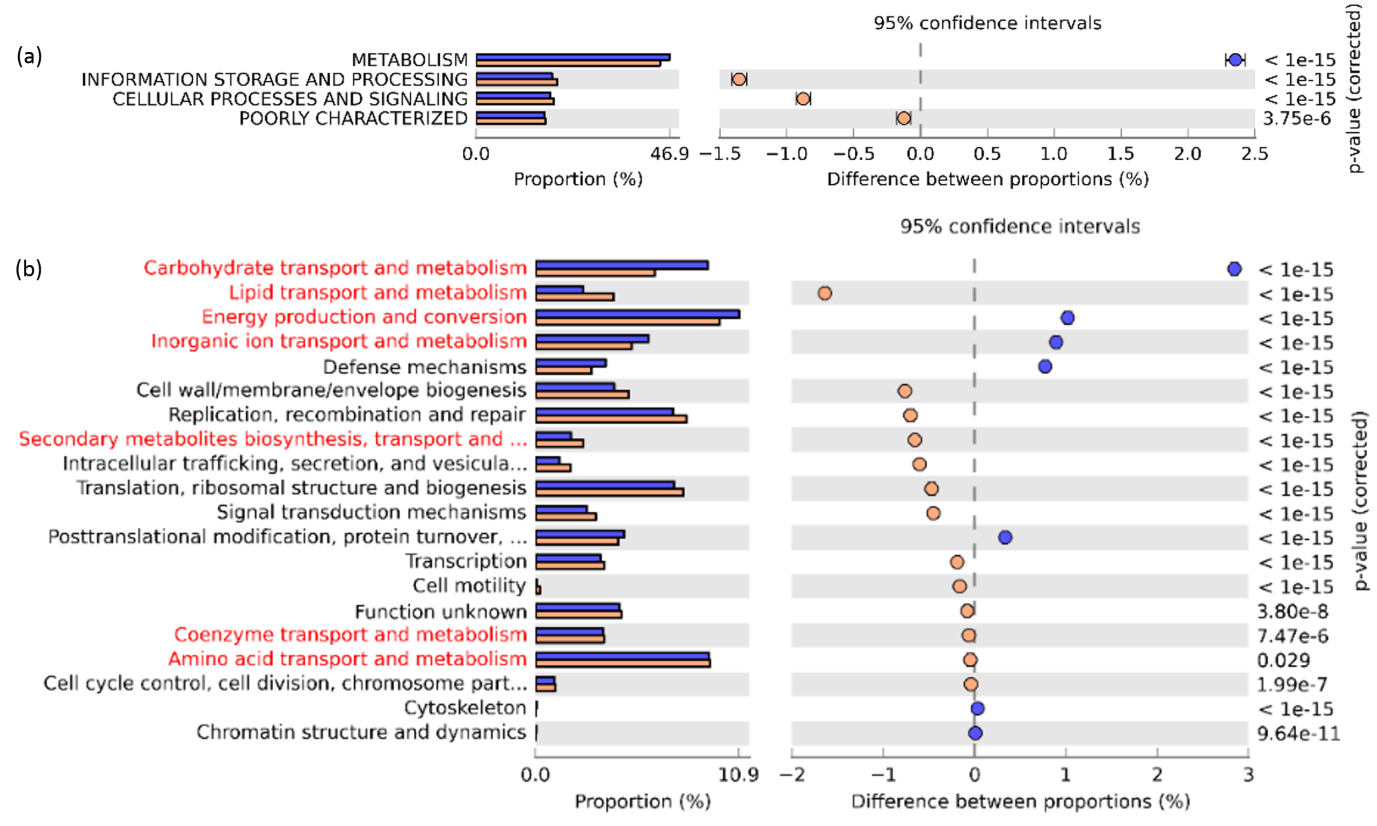
**

Figure S3. COG (a) level 1 and (b) level 2 categories that differed significantly between the GZ (blue) and SWH (yellow) metagenomes. Significance was determined by Fisher’s exact test with Storey’s FDR correction for multiple comparisons, with corrected p < 0.05 considered significant, and differences between two samples with proportions > 0.01 were selected. Functions within the category ‘Metabolism’ are labeled in red.


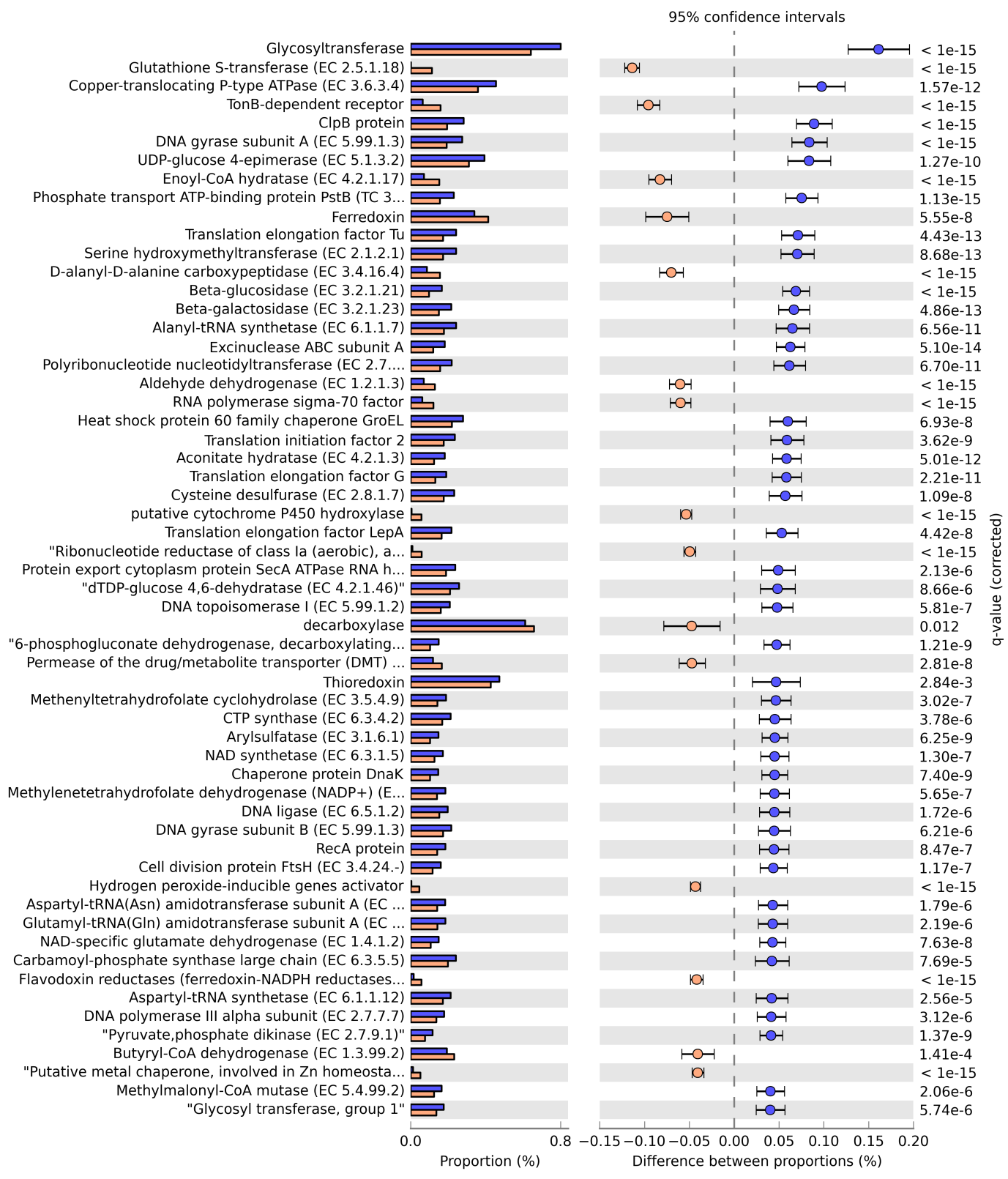


Figure S4. Significant and large functional differences between GZ (blue) and SWH (yellow) level 4 SEED subsystems. Significance was determined by Fisher’s exact test with Storey’s FDR correction for multiple comparisons, with corrected p < 0.05 considered significant. Only subsystems with differences between proportions > 0.04 (i.e. considered large effect) are shown.
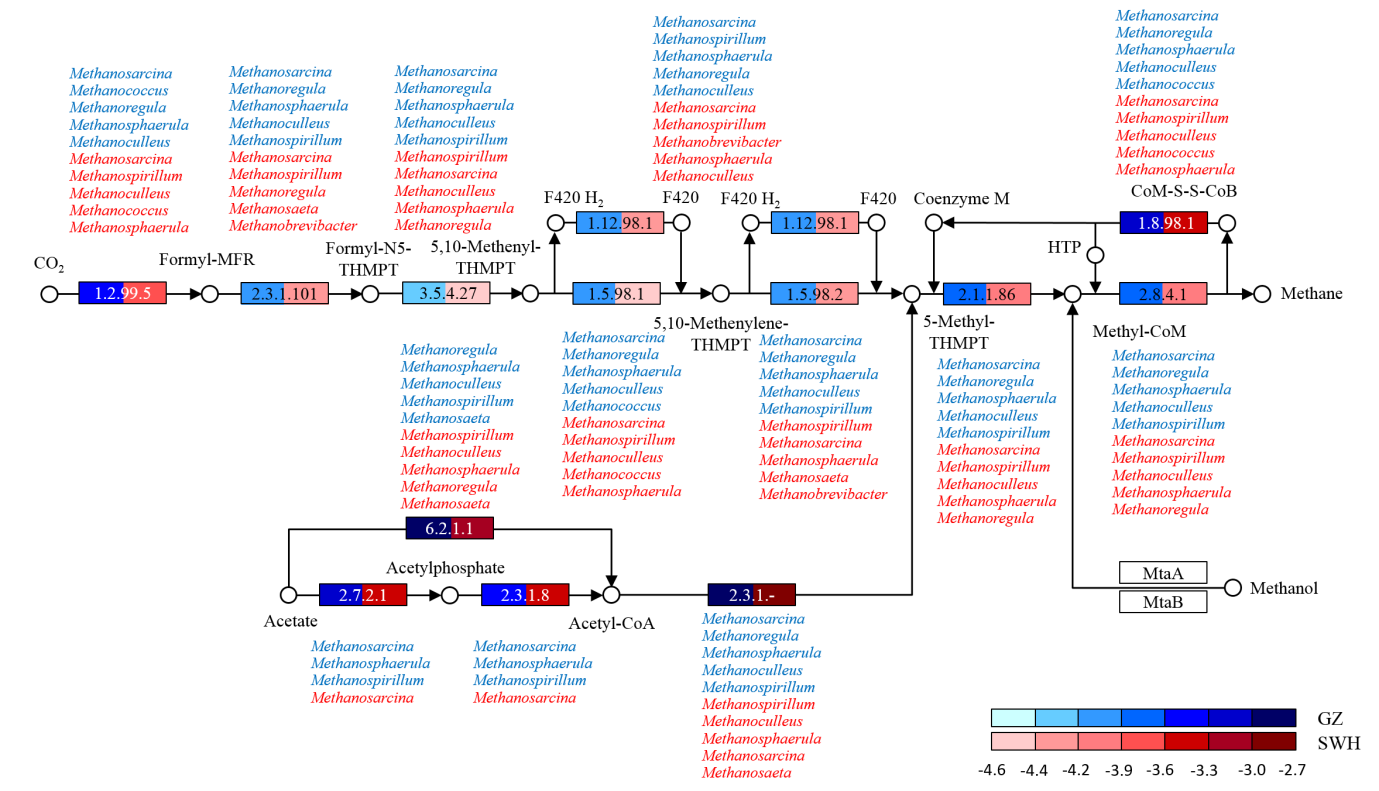


Figure S5. Mapping of genera detected in the GZ and SWH metagenomes to functional steps in the methanogenesis pathway. Numbered rectangles represent enzymes, and the log_10_-transformed relative abundance of each enzyme in each metagenome is represented by the shade of blue and red for GZ and SWH, respectively. Beside each enzyme is listed the key methanogenic genera (ordered based on the abundance of the available genus) mapped to that enzyme as determined by annotation of reads against the SEED database, with blue and red text representing the presence of that genus in GZ and SWH, respectively. The methanogenesis pathway was constructed based on representative pathways in the KEGG, MetaCyc and BRENDA databases.


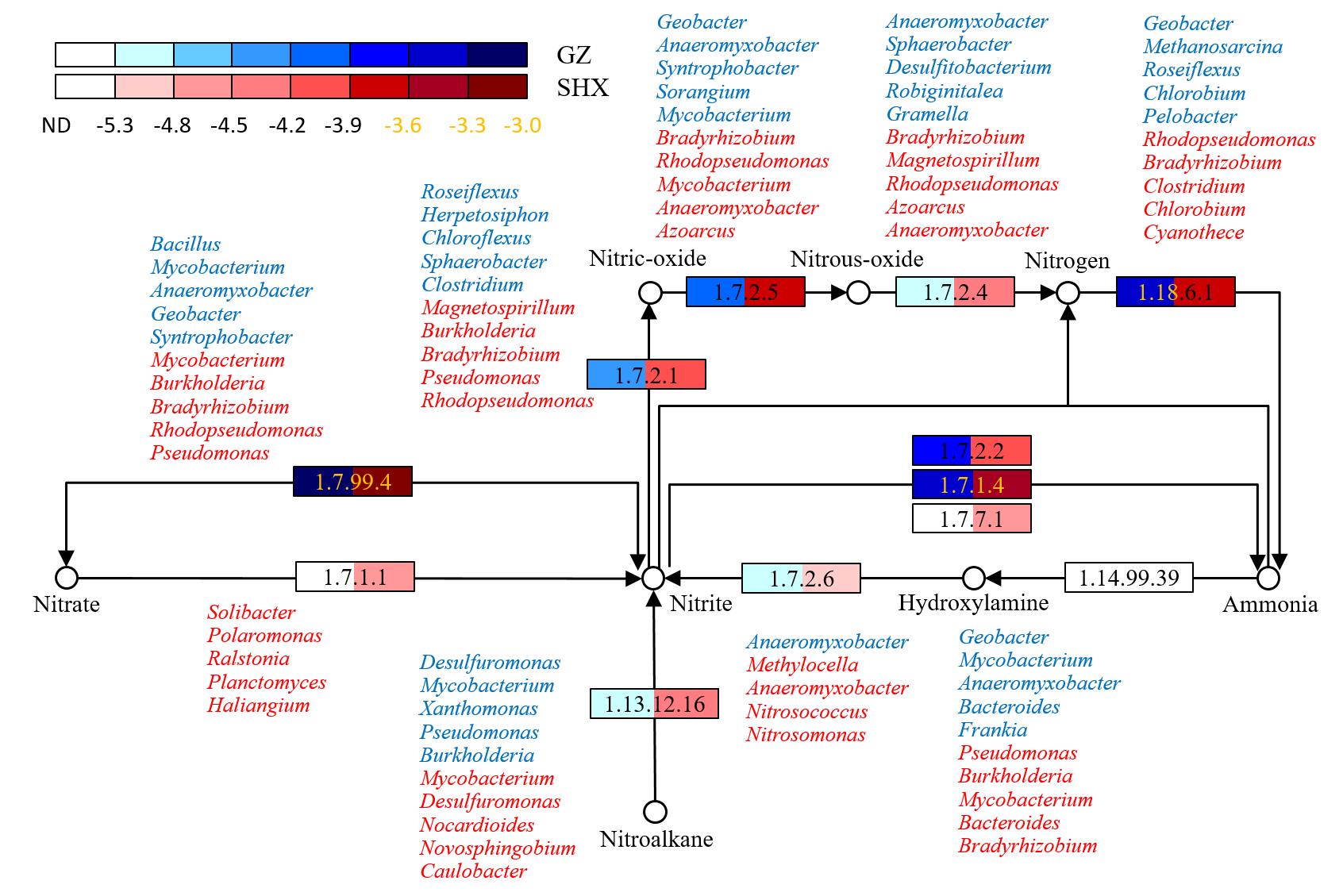


Figure S6. Mapping of genera detected in the GZ and SWH metagenomes to functional steps in the nitrogen removal pathway. Numbered rectangles represent enzymes, and the log_10_-transformed relative abundance of each enzyme in each metagenome is represented by the shade of blue and red for GZ and SWH, respectively. Beside each enzyme is listed the key methanogenic genera (ordered based on the abundance of the available genus) mapped to that enzyme based on annotation of reads against the SEED database, with blue and red text representing the presence of that genus in GZ and SWH, respectively. The methanogenesis pathway was constructed based on representative pathways in the KEGG, MetaCyc and BRENDA databases.


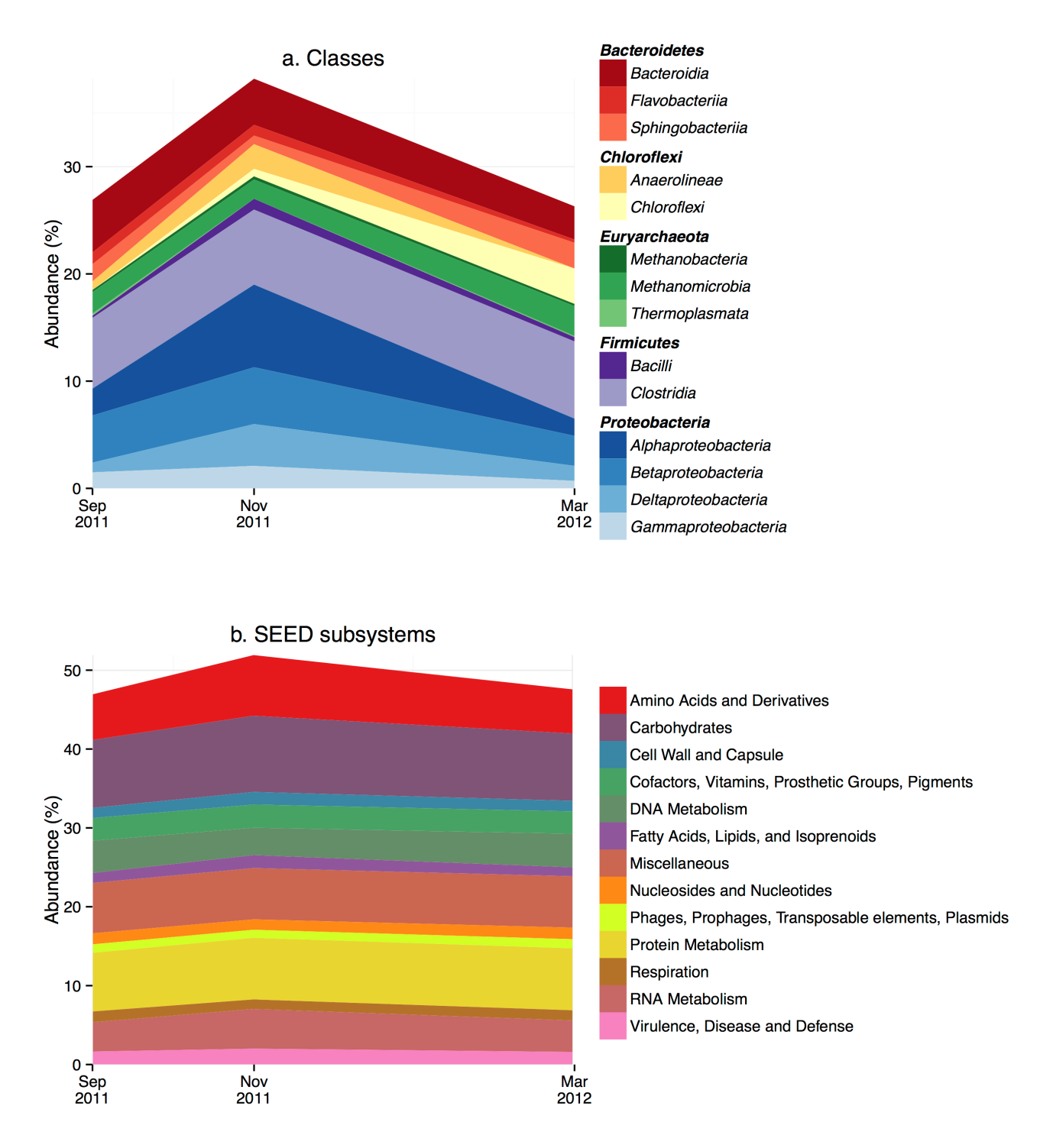


Figure S7. Most abundant (a) classes and (b) SEED subsystems (level 2) in SWH metagenomes sampled at different times. The samples collected in September 2011 and March 2012 are from a previous study ([Yang et al., 2014](#_ENREF_5)), while the sample collected in November 2011 is from this study.

**References**

Lv, X. M., Shao, M. F., Li, C. L., Li, J., Gao, X. L., Sun, F. Y. (2014). A Comparative Study of the Bacterial Community in Denitrifying and Traditional Enhanced Biological Phosphorus Removal Processes. *Microbes Environ.* 29, 261-268. doi: 10.1264/jsme2.ME13132

Schluter, A., Bekel, T., Diaz, N. N., Dondrup, M., Eichenlaub, R., Gartemann, K. H., et al. (2008). The metagenome of a biogas-producing microbial community of a production-scale biogas plant fermenter analysed by the 454-pyrosequencing technology. *J. Biotechnol.* 136, 77-90. doi: 10.1016/j.jbiotec.2008.05.008

Wilkins, D., Lu, X. Y., Shen, Z., Chen, J., Lee, P. K. H. (2015). Pyrosequencing of *mcrA* and archaeal 16S rRNA genes reveals diversity and substrate preferences of methanogen communities in anaerobic digesters. *Appl. Environ. Microbiol.* 81, 604-613. doi: 10.1128/AEM.02566-14

Xia, Y., Ju, F., Fang, H. H. P., Zhang, T. (2013). Mining of Novel Thermo-Stable Cellulolytic Genes from a Thermophilic Cellulose-Degrading Consortium by Metagenomics. *PLoS One* 8, e53779. doi: 10.1371/journal.pone.0053779

Yang, Y., Yu, K., Xia, Y., Lau, F. T., Tang, D. T., Fung, W. C., et al. (2014). Metagenomic analysis of sludge from full-scale anaerobic digesters operated in municipal wastewater treatment plants. *Appl. Microbiol. Biotechnol.* 98, 5709-5718. doi: 10.1007/s00253-014-5648-0
